# Antifungal Peptides

**DOI:** 10.3390/jof7060437

**Published:** 2021-05-31

**Authors:** Gill Diamond

**Affiliations:** Department of Oral Immunology and Infectious Diseases, University of Louisville, Louisville, KY 40202, USA; gill.diamond@louisville.edu

Fungal infections represent an increasing public health crisis. Not only is there the constant increase in resistance to existing antifungal drugs but there is also the emergence of new pathogenic fungi, most notably the multi-drug-resistant *Candida auris*. As the most recently developed class of antifungal drugs, the echinocandins, were unveiled nearly 30 years ago, there is obviously a great need for new therapies. Antimicrobial peptides (AMPs), also called host defense peptides, have been studied for over 40 years as potential therapeutic agents for microbial infections. While the primary focus of research was initially against bacterial pathogens, both the discovery of strongly antifungal peptides, especially in plants and in human saliva, has allowed for the expansion of this research into their antifungal activity. In this Special Issue, numerous peptide classes, both naturally occurring and synthetic, are examined experimentally for their activity in vitro and in vivo against a variety of fungal pathogens. One striking observation is the wide variety of natural sources for the peptides. Ciociola et al. [1] describe the in vitro and in vivo activity of a synthetic peptide that mimics a yeast killer toxin against *C. albicans*. Further expanding on this concept, Woodburn et al. [2] demonstrated potent activity of novel peptides whose sequences were designed based on the β-defensin structure. Activity was shown against a wide variety of fungal pathogens, from *Candida, Aspergillus, Absidia, Fusarium* and *Mucor* species. As plants represent a major source of antifungal peptides, Bleackley et al. [3] focus on a novel subset of plant defensin sequences that are enriched in histidine residues. These peptides exhibit potent activity against both *Fusarium* and *Candida* species. A novel source of new antifungal peptides are filamentous fungi. Holzknecht et al. [4] demonstrate the potent activity of a peptide from *Penicillium chrysogenum* against *C. albicans*, which acts through both the membrane disruption mechanism common to most AMPs and intracellularly. In contrast to most AMPs, the human saliva-derived peptide, histatin 5, is highly specific for its activity against *Candida* species. Furthermore, its mechanism of action differs from most peptides, being primarily intracellular. Norris et al. [5] demonstrate a novel mechanism in the presence of zinc that involves the fungal membrane, expanding the numbers of potential pathogenic targets. Finally, Ryan et al. [6] describe a novel method for in vivo testing of antifungal drugs, using small molecule mimics of AMPs in a mouse strain deficient in a β-defensin. Three review articles are also published in this issue, providing the most recent literature on the activity of peptides and their potential as antifungal drugs. Basso et al. [7] discuss a wide variety of AMPs, both naturally occurring and synthetic, that exhibit potent antifungal activity, and which could serve as a template for new drugs. The article by Su et al. [8] reviews the most recent literature on the class of potent antifungal peptides from plants known as Snakins. Finally, Candido et al. [9] discuss the activity of echinocandins and their potential use in synergy with other antifungal drugs against *C. auris*. We would like to thank all of the authors and reviewers who contributed to this issue, and we hope the information will contribute to the further development of important new antifungal therapies.
